# Comparative image quality evaluation of unenhanced postmortem Photon-Counting CT and Energy-Integrating CT

**DOI:** 10.1007/s12024-025-01118-0

**Published:** 2025-12-06

**Authors:** Conny Hartmann, Magda Roidou, Paolo Lombardo, Samira Kessler, Hendrik von Tengg-Kobligk, Thomas Ruder, Stefanie Garni, Peter Cossmann, Sarah Heinze, Wolf-Dieter Zech

**Affiliations:** 1 Department of Forensic Medicine and Imaging, Institute of Forensic Medicine, University of Bern, Bern, Switzerland; 2 Department of Diagnostic, Interventional and Pediatric Radiology, Inselspital, Bern University Hospital, University of Bern, Bern, Switzerland; 3 MedTechConsulting Cossmann GmbH, Zug, 6300 Switzerland; 4 Diagnostic and Research Institute of Forensic Medicine, Medical University of Graz, Graz, Austria; 5 Fachärztin Forensische Medizin Institut für Rechtsmedizin, Murtenstrasse 26, 3008 Bern, Switzerland

**Keywords:** Post-mortem imaging, Photon-counting detector CT, Post-mortem photon-counting detector CT, Forensic radiology, Image quality

## Abstract

This study evaluates the image quality of postmortem photon-counting computed tomography (PMPCCT) compared to conventional postmortem energy-integrating computed tomography (PMCT) for a protocol adapted to post-mortem imaging. The focus lies on objective image quality parameters such as noise, signal-to-noise ratio, and contrast-to-noise ratio, supplemented by subjective image quality evaluations. A Chest Phantom N1 and one decedent were scanned using PMCT (Siemens SOMATOM^®^ X-Cite; slice thicknesses of 1 mm and 0.5 mm; Br40, Br60) and PMPCCT (Siemens NAEOTOM Alpha.peak^®^; slice thicknesses of 1 mm, 0.4 mm, and 0.2 mm; Br40, Br60). Image quality parameters were computed for regions of interest. In addition, two radiologists conducted subjective image quality evaluations (noise, overall image quality, sharpness, bone details, contour visibility, and artifact formation) using a modified Likert scale. The overall findings were mixed, with PMPCCT potentially demonstrating an advantage over PMCT in terms of SNR and CNR, particularly at moderate slice thicknesses of 1 mm and 0.4 mm. The results obtained from the phantom exhibit in some cases considerable differences compared to those from the decedent, so that model studies can hardly be transferred to reality without scrutiny. PMPCCT outperformed PMCT in subjective assessments of overall image quality, sharpness, and bone detail, particularly with Br40 reconstruction kernels. PMPCCT may demonstrate advantages in objective and subjective image quality, with improvements in soft tissue imaging, sharpness, and bone detail at moderate slice thicknesses. These results may suggest PMPCCTs promising potential in forensic imaging. Further studies are needed to investigate and optimize its utility in postmortem settings.

## Introduction

Over the past two decades, postmortem energy-integrating computed tomography (PMCT) has become a vital adjunct to traditional autopsy in forensic medicine, offering non-invasive imaging techniques to enhance postmortem diagnostics [1–4]. The advent of photon-counting computed tomography (PCCT) represents a significant technological advancement with the potential to revolutionize both clinical and forensic post-mortem imaging [5–8]. Unlike conventional energy-integrating detector CT systems (CT), PCCT uses photon-counting detectors to register and analyze individual X-ray photons, including their energy levels [8–12]. This approach enables ultra-high spatial resolution (up to 0.2 mm), reduced image noise, minimized image artifacts, and intrinsic spectral or multi-energy imaging capabilities, surpassing the limitations of CT [13–29].

In clinical radiology, PCCT has demonstrated substantial potential, offering improved diagnostic detail in cardiovascular [17, 26, 27], chest [20, 21], and musculoskeletal imaging [28, 29]. The ability of this technology to provide ultra-high spatial resolution and to perform advanced material characterization [8, 13, 17, 18, 30] and quantitative imaging [24, 25] suggests it could also deliver significant advantages in forensic post-mortem applications. In particular, its ultra-high-resolution capabilities [22, 23] could aid detecting forensically relevant small fractures, vascular injuries, or foreign objects, such as projectiles, knife or glass fragments, drug body packs, or implanted medical devices [8, 9, 25]. The intrinsic spectral capabilities offer the possibility for characterization and differentiation of tissues and forensically relevant foreign bodies [13–15, 17, 18, 30, 31].

Despite its potential, the application of PCCT in postmortem imaging remains largely unexplored, with scarce documented research on its diagnostic value in post-mortem forensic investigations. Unlike clinical CT, post-mortem CT typically uses significantly higher radiation doses and/or changes in the kilovoltage setting with appropriately adapted CT protocols [8, 32]. The specific effects of higher radiation doses on image quality in PMPCCT compared to PMCT remain to be investigated. Thus, this study aims to assess the objective and subjective image quality of non-spectral PMPCCT compared to PMCT for protocols adapted to post-mortem imaging.

## Materials and methods

### Study design

This prospective study compared PMCT to PMPCCT in one deceased human body and in a radiological phantom, assessing both objective and subjective image quality parameters. The study was conducted in compliance with the ethical standards of the Declaration of Helsinki and approved by the Cantonal Ethics Committee of Bern (reference number 2023 − 00460).

**Fig. 1:**
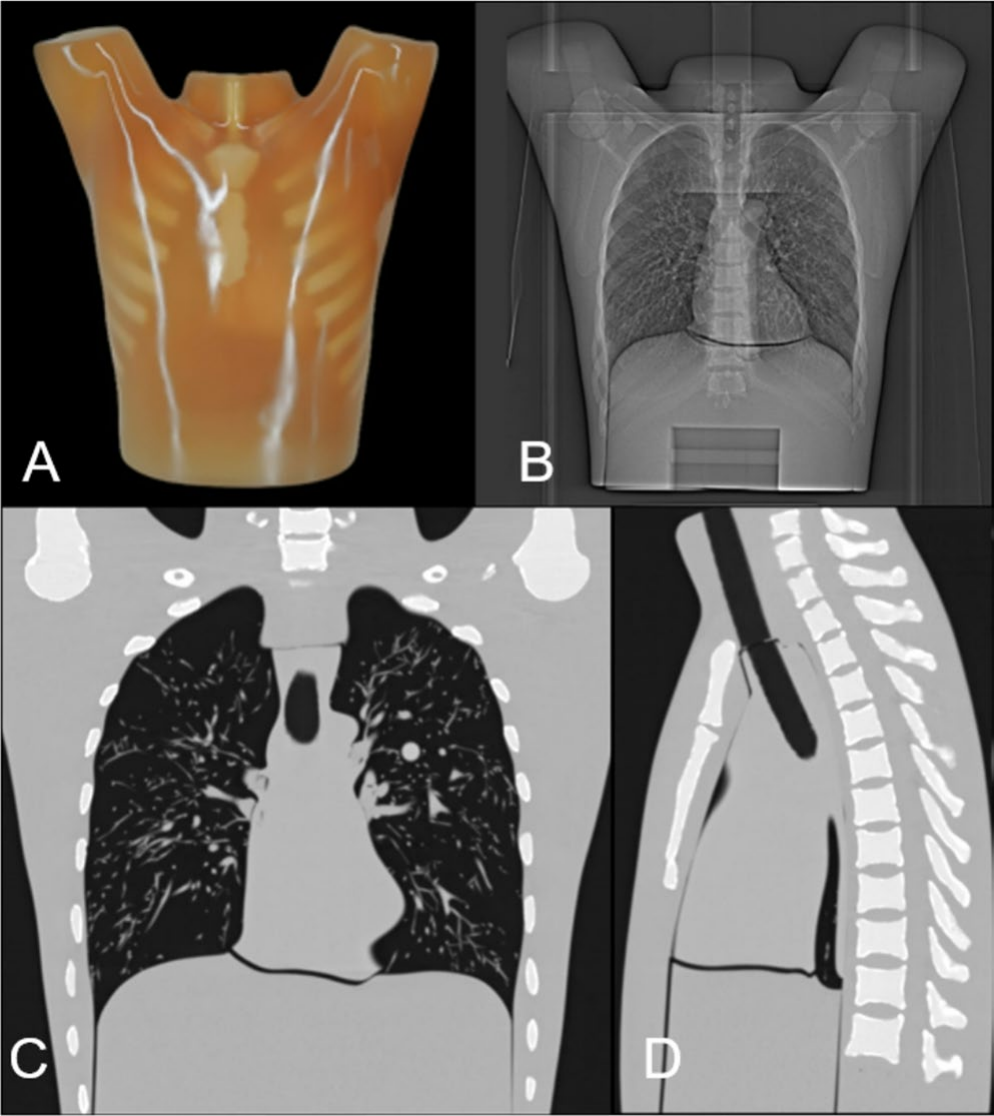
Multipurpose Chest Phantom N1: Main body with synthetic bones embedded (**A**). Topogramm (**B**), coronal (**C**) and sagittal (**D**) view in a CT scan of the phantom

The study involved a 40-year-old male (81 kg, 187 cm, BMI 23,2 kg/m^2^) who died of acute cardiac failure. His medical history included an ICD implantation for myocarditis, enabling metal artifact analysis. Additionally, scans were performed using the Multipurpose Chest Phantom N1 (Kyoto Kagaku Co. Ltd), a life-size male torso model designed for imaging applications (Fig. 1). Three spherical inserts simulating lung nodules with varieties of Hounsfield numbers (approx. −800, −630 and + 100) were placed into the phantom lung. The phantom chest wall consisted of both synthetic soft tissues and synthetic bones with imaging properties that are modeled on clinical CT-data to ensure realistic radiologic imaging characteristics and X-ray absorption properties. Key anatomical features include a mediastinum, spatially positioned pulmonary vessels, and a removable abdominal section (diaphragm block). Materials: soft tissue = urethane-based resin (specific gravity: 1.06), synthetic bone = Epoxy resin (specific gravity: 1.31). The phantom was included to enable standardized and reproducible measurements under controlled conditions, while the decedent scan reflected real-world postmortem imaging scenarios.

PMCT imaging was performed at the Department of Forensic Medicine and Imaging of the Institute of Forensic Medicine Bern using a Siemens SOMATOM X-Cite CT scanner. PMPCCT imaging was performed at the Department of Diagnostic, Interventional and Pediatric Radiology at the University Hospital of Bern using a Siemens NAEOTOM Alpha.peak photon-counting CT scanner. The phantom was scanned on the same day using both PMPCCT and PMCT, while the decedent was scanned with PMPCCT two days after its PMCT scan due to logistical constraints of the local service systems. The body was therefore stored at 4 °C to minimize decomposition prior to imaging. While a minor influence of the postmortem interval and body temperature on imaging cannot be fully excluded, major effects on the evaluated tissues were considered unlikely.

### Imaging protocols

Table 1 Shows the PMCT and PMPCCT protocols applied in this study. Decedent scans included thorax and abdomen, while the Phantom was scanned completely. PMCT images were reconstructed at 1.0 mm and 0.5 mm slice thickness with a 512 × 512 matrix using BR40 and BR60 kernels, in accordance with previous postmortem imaging recommendations [8, 32]. PMPCCT images were reconstructed at 1.0 mm, 0.4 mm, and 0.2 mm slice thickness with a 1024 × 1024 matrix using the same kernels; the thinner reconstructions were included to investigate the ultra-high-resolution potential of photon-counting CT. For both systems, reconstruction settings were chosen according to standard usage to achieve optimal image quality and to reflect typical clinical and forensic practice.

**Table 1:** Imaging parameters for PMPCCT and PMCT

	Acquisition parameters							Reconstruction parameters					
Scanner	Scan Object	Collimation (mm)	kV	Eff. mAs	CTDI mGy	Pitch	Rotation Time	Slice Thickness	Increment	Matrix	Kernel	FoV	Reconstruction level
PMCT	Decedent	120 × 0.2	140	500	46	0.25	2000 ms	1 mm 0.5 mm	0.7 mm 0.25 mm	512	BR40/BR60	422	Admire 3
PMPCCT	Decedent	120 × 0.2	140	500	53	0.25	2000 ms	1 mm 0.4 mm 0.2 mm	0.5 mm 0.2 mm 0.1 mm	1024	BR40/BR60	422	Quantum Interactive 3
PMCT	Phantom	120 × 0.2	140	200	24	0.25	2000 ms	1 mm 0.5 mm	0.7 mm 0.25 mm	512	BR40/BR60	350	Admire 3
PMPCCT	Phantom	120 × 0.2	140	200	23	0.25	2000 ms	1 mm 0.4 mm 0.2 mm	0.5 mm 0.2 mm 0.1 mm	1024	BR40/BR60	350	Quantum Interactive 3

### Objective image quality analysis

**Fig. 2:**
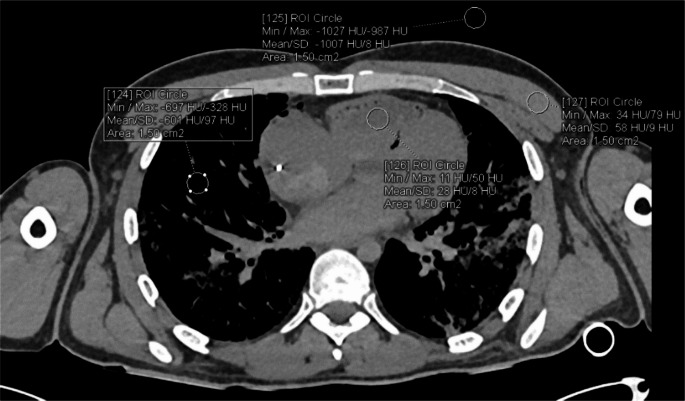
Exemplary density measurement on the decedent in PMCT, slice thickness 0.5 mm, Br40 kernel. ROI measurements (mean density values) from left to right: lung = −601 HU, blood = 28 HU, background (air) = −1007 HU, muscle = 58 HU

**Fig. 3:**
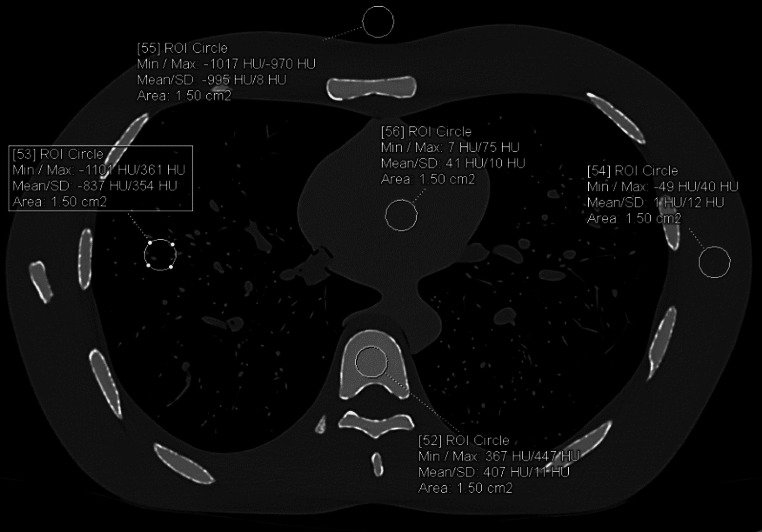
Exemplary density measurement on the phantom in PMPCCT, slice thickness 0.4 mm, Br 60 kernel. ROI measurements (mean density values) at the top, right, bottom, left and center: background (air) = −995 HU, chest wall = 1 HU, vertebral body = 407 HU, lung = −837 HU, blood = 41 HU

Objective image quality in this study was considered in terms of quantitative parameters, specifically noise, signal-to-noise ratio (SNR), and contrast-to-noise ratio (CNR). Quantitative measurements as means and standard deviations of Hounsfield Units (HU) were performed using a syngo.via workstation (Siemens, Erlangen, Germany). Regions of interest (ROIs) in both the decedent and the phantom were defined, according to first different and secondly similar attenuation values to test the full possible potential of the PMDCDCT technique. In the decedent, ROIs with a diameter of 1.5 cm were measured in the vertebral body, lung, blood in the right ventricle, muscle, and liver (Fig. 2). In the phantom, ROIs of the same diameter were placed in the vertebral body, chest wall, mediastinum, and diaphragm block. Additionally, three spherical inserts in the phantom lung were analyzed using ROIs with a diameter of 0.5 cm (Fig. 3). The ROIs were localized at the same positions in PMCCT and PMCT using the copy-paste function of the syngo.via workstation within the same imaging method and by visually aligning anatomical landmarks of the body between the two imaging methods. Measurements were conducted by a forensic pathology resident under the supervision of a board-certified forensic pathologist with 15 years of experience in postmortem imaging. All measurements were taken across five consecutive slices for each slice thickness and reconstruction kernel, and subsequently averaged.

Noise, SNR, CNR were calculated for each ROI according to the following definitions:

- Noise: standard deviation of the Hounsfield Units comprising the background noise.
- SNR calculated as: $\:\frac{\text{Mean HU}\left(\mathrm{ROI}\right)}{\mathrm{SD}\text{ }\text{(Standard deviation) HU (Background)}}$
- CNR calculated as: $\:\frac{\text{Mean HU}\:\left(\mathrm{ROI}\right)\text{ - Mean HU (Background)}}{\text{SD HU (Background)}}$

Percent changes in noise, SNR, and CNR between PMPCCT and PMCT were calculated for all slice thicknesses (1 mm (PMPCCT) vs. 1 mm (PMCT), 0.4 mm (PMPCCT) vs. 0.5 mm (PMCT), 0.2 mm (PMPCCT) vs. 0.5 mm (PMCT)) using the formula: $\frac{\left(\mathrm{Value}\;1:\;\mathrm{Value}\;2\right)^\ast100\%}{\mathrm{Value}\;2}$.

The PMCT was chosen as the reference method, so that the HU measurement of the PMCT corresponds to the Values 2.

### Subjective image quality assessment

Subjective image quality in this study was considered in terms of perceptual parameters relevant to forensic imaging, including noise impression, overall image impression, sharpness, bone detail, contour visibility, and artifact formation. Two independent readers (a board-certified radiologist and a radiology resident), both experienced in postmortem imaging, independently assessed subjective image quality to capture potential differences related to experience level. Consensus reading was deliberately avoided to preserve interobserver variability. For Br40 reconstructions, soft tissue windows (approx. WW 350–400 HU/WL ~ 40 HU) were applied, and for Br60 reconstructions, bone windows (approx. WW 1500–2000 HU/WL ~ 300–400 HU), corresponding to standard default settings of the syngo.via workstation. A modified 5-point Likert scale (1 = best rating, 5 = worst rating across all parameters) was applied to evaluate noise, overall image quality, sharpness, bone details, contour visibility (spherical inserts in the phantom or lymph nodes in the decedent), and artifact formation caused by the ICD catheter in the decedent. Ratings were recorded for each reconstruction kernel and slice thickness.

## Results

The detailed data for SNR, CNR and Noise are presented in Table 2, while the calculated percent change values can be viewed in the appendix (Table 4).

**Table 2:** Overview of the signal-to-noise ratios (SNR), contrast-to-noise ratios (CNR) and noise between PMPCCT and PMCT across various slice thicknesses, reconstruction kernels, and tissue types (values rounded to whole numbers if greater than 1, values for spherical inserts not shown)

Phantom					
	**PMPCCT**			**PMCT**	
Slice thickness	**1 mm**	**0.4 mm**	**0.2 mm**	**1 mm**	**0.5 mm**
SNR	**Br40/Br60**	**Br40/Br60**	**Br40/Br60**	**Br40/Br60**	**Br40/Br60**
Vertebral Body	172/57	205/57	104/34	191/49	127/37
Lung	−343/−113	−411/−113	−206/−68	−419/−113	−278/−85
Chest wall	0,3/0,08	0,6/0,1	0,6/0,2	5/1	3/1
Mediastinum	16/5	20/5	9,9/3,3	20/5	13/4
Diaphragm block	−0,07/0	0/0	0/0	1/0,3	0,8/0,2
CNR	**Br40/Br60**	**Br40/Br60**	**Br40/Br60**	**Br40/Br60**	**Br40/Br60**
Vertebral Body	588/196	704/195	353/116	689/177	460/133
Lung	73/25	88/26	44/14	80/15	54/11
Chest wall	417/139	500/139	250/82	504/130	336/97
Mediastinum	433/144	519/144	260/85	519/134	346/100
Diaphragm block	333/98	277/80	172/57	335/85	251/61
Noise	**Br40/Br60**	**Br40/Br60**	**Br40/Br60**	**Br40/Br60**	**Br40/Br60**
Background	3/8	2/8	4/13	2/8	3/11
Decedent					
	**PMPCCT**			**PMCT**	
Slice thickness	**1 mm**	**0.4 mm**	**0.2 mm**	**1 mm**	**0.5 mm**
SNR	**Br40/Br60**	**Br40/Br60**	**Br40/Br60**	**Br40/Br60**	**Br40/Br60**
Vertebral Body	81/30	64/18	54/17	60/28	42/21
Lung	−102/−32	−66/−20	−51/−18	−138/−71	−104/−52
Muscle	16/5	11/3	9/3	13/6	10/5
Blood	11/3	7/2	6/2	7/3	5/2
Liver	15/6	12/3	10/3	9/4	7/4
CNR	**Br40/Br60**	**Br40/Br60**	**Br40/Br60**	**Br40/Br60**	**Br40/Br60**
Vertebral Body	414/155	314/91	271/89	354/167	242/125
Lung	175/55	119/32	100/33	90/46	70/34
Muscle	294/93	196/55	160/54	241/123	183/90
Blood	288/91	192/54	157/53	235/120	178/88
Liver	265/96	211/58	177/56	188/97	150/77
Noise	**Br40/Br60**	**Br40/Br60**	**Br40/Br60**	**Br40/Br60**	**Br40/Br60**
Background	4/11	5/18	6/18	4/9	6/12

### PMPCCT vs. PMCT in phantom, Br40

For Br40, PMPCCT generally yielded lower SNR and CNR values than PMCT, with higher noise levels. However, in the 0.4 mm vs. 0.5 mm setting, PMPCCT outperformed PMCT, showing higher SNR (vertebral body: +60.9%, lung: +47.6%, mediastinum: +47%) and CNR across all investigated tissues, with reduced noise.

### PMPCCT vs. PMCT in decedent, Br40

Across all slice thickness comparisons, PMPCCT generally showed higher SNR, CNR, and lower noise than PMCT. Exceptions were SNR in lung tissue, where PMCT outperformed PMPCCT, as well as SNR and CNR in muscle and CNR in blood, each in the 0.2 mm vs. 0.5 mm setting. Across all slice thickness comparisons, PMPCCT generally showed higher SNR and CNR as well as lower noise than PMCT. Exceptions were SNR in lung tissue, where PMCT consistently outperformed PMPCCT, and SNR and CNR in muscle in the 0.2 mm vs. 0.5 mm setting and CNR in blood in the 0.2 mm vs. 0.5 mm setting.

### PMPCCT vs. PMCT in phantom, Br60

At Br60, SNR varied without clear trends. PMPCCT outperformed in the vertebral body (+ 17.4%) and mediastinum (+ 3%) but trailed PMCT in the chest wall (−94.1%) and liver (−100%). CNR was mostly higher in PMPCCT, except in the 0.2 mm vs. 0.5 mm setting, where PMCT performed better for most tissues. Noise was generally lower in PMPCCT, except in the 0.2 mm vs. 0.5 mm comparison.

### PMPCCT vs. PMCT in decedent, Br60

At Br60, SNR and CNR were generally higher in PMCT, except in the 1 mm vs. 1 mm setting, where SNR was higher in the vertebral body (+ 9.2%) and liver (+ 24.1%), and CNR was higher in the lung (+ 21.4%) in PMPCCT. Noise was consistently lower in PMCT.

**Fig. 4:**
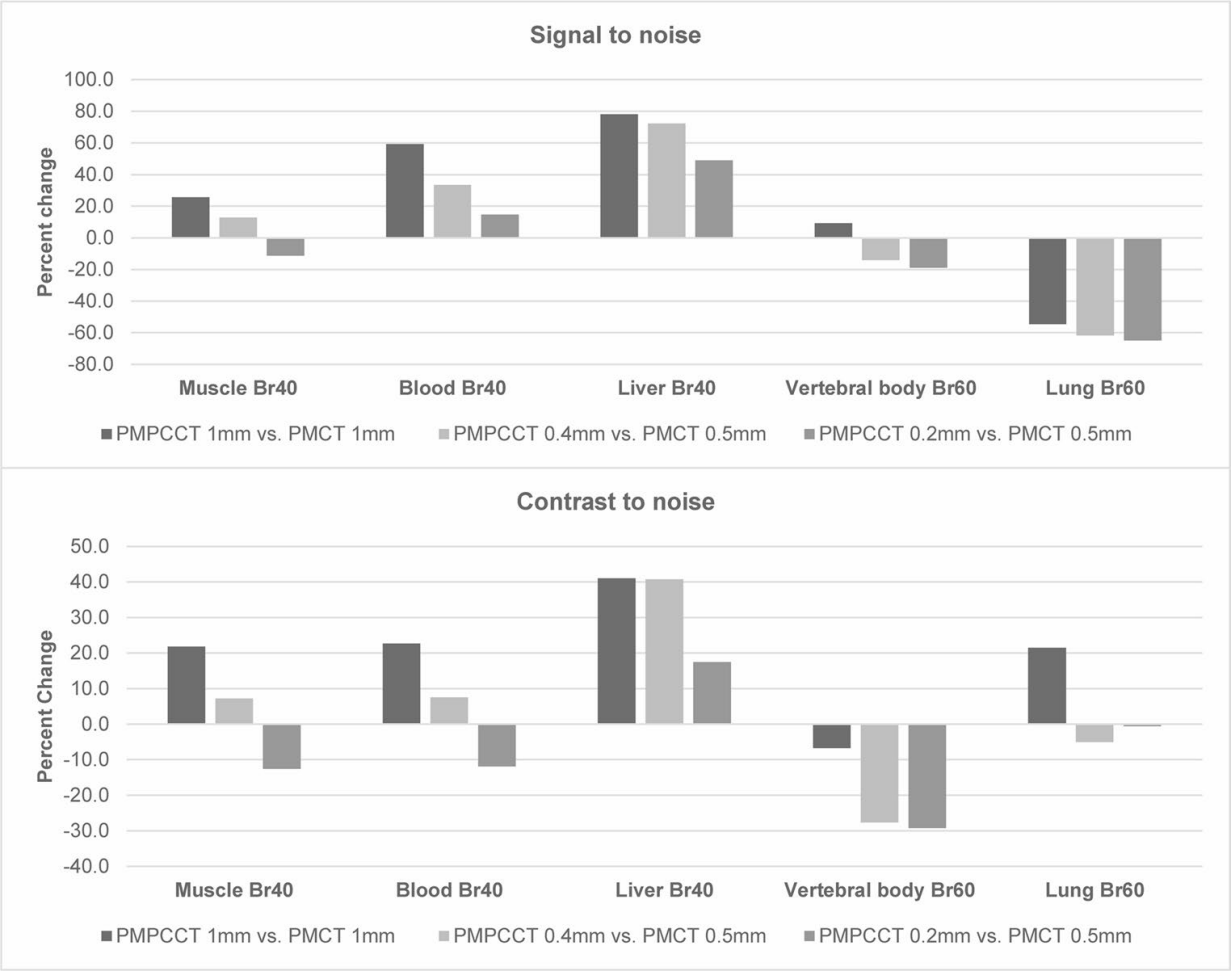
Illustration of the percent change in signal-to-noise (upper chart) and contrast-to-noise (lower chart) ratios between PMPCCT and PMCT for the decedent for different slice thickness pairs (1 mm vs. 1 mm, 0.4 mm vs. 0.5 mm and 0.2 mm vs. 0.5 mm). Positive bars indicate higher PMPCCT values while negative bars indicate lower values. Note the different reconstruction kernels

**Fig. 5:**
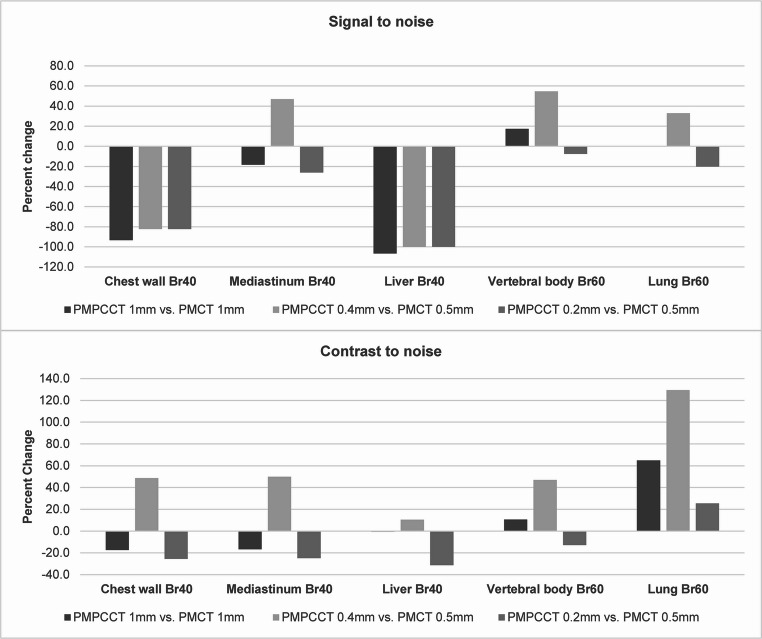
Illustration of the percent change in signal-to-noise (upper chart) and contrast-to-noise (lower chart) ratios between PMPCCT and PMCT for the phantom for different slice thickness pairs (1 mm vs. 1 mm, 0.4 mm vs. 0.5 mm and 0.2 mm vs. 0.5 mm). Positive bars indicate superiority of PMPCCT while negative bars indicate inferiority. Note the different reconstruction kernels

The bar charts (Fig. 4) show the percent change values of the SNR and CNR values in the decedent graphically for different slice thickness pairs (1 mm vs. 1 mm, 0.4 mm vs. 0.5 mm and 0.2 mm vs. 0.5 mm), whereby only the Br40 reconstruction kernels for soft tissue types and the Br60 reconstruction kernels for bone and lung are shown. Bar charts showing the percent change values of the SNR and CNR values in the phantom can be viewed in the appendix (Figure 5).

### Results of subjective image quality assessment

Overall, PMPCT was consistently rated higher than PMCT in terms of image quality, sharpness, and bone detail, with a slight advantage in noise reduction at larger slice thicknesses. Detailed ratings are shown in Table 3.

**Table 3:** Subjective evaluation of image quality for PMPCCT and PMCT across different reconstruction kernels and slice thicknesses. The assessed parameters were rated using a modified 5-point likert scales: noise and artifact formation: 1 = Minimal, 2 = Few, 3 = Noticeable, 4 = Significant, 5 = Severe; overall image quality, Sharpness, bone details, and contour visibility: 1 = Very good, 2 = Good, 3 = Moderate, 4 = Poor, 5 = Very poor

Phantom										
Kernel	**Br40**					**Br60**				
	**PMPCCT**			**PMCT**		**PMPCCT**			**PMCT**	
Slice thickness	**1**	**0.4**	**0.2**	**1**	**0.5**	**1**	**0**,**4**	**0.2**	**1**	**0.5**
Radiology Specialist/Resident (S/R)	**S/R**	**S/R**	**S/R**	**S/R**	**S/R**	**S/R**	**S/R**	**S/R**	**S/R**	**S/R**
Noise	4/2	2/4	2/5	4/2	4/3	1/3	2/4	1/5	1/2	1/3
Overall image quality	4/2	4/2	3/2	5/3	5/3	2/2	1/2	1/3	2/3	2/3
Sharpness	4/3	4/2	4/2	4/3	5/2	2/2	1/1	1/1	2/3	2/2
Bone details	4/3	4/2	4/2	5/3	4/2	2/2	1/1	1/1	3/3	1/2
Contour visibility (spherical inserts)	3/2	3/2	2/2	2/3	3/2	1/1	1/1	1/1	1/3	1/2
Decedent										
Kernel	**Br40**					**Br60**				
	**PMPCCT**			**PMCT**		**PMPCCT**			**PMCT**	
Slice thickness (in mm)	**1**	**0.4**	**0.2**	**1**	**0.5**	**1**	**0.4**	**0.2**	**1**	**0.5**
Radiology Specialist/Resident (S/R)	**S/R**	**S/R**	**S/R**	**S/R**	**S/R**	**S/R**	**S/R**	**S/R**	**S/R**	**S/R**
Noise	1/2	1/3	1/4	2/2	1/3	2/3	1/4	1/5	2/3	2/4
Overall image quality	2/2	2/1	1/2	2/3	3/3	1/2	1/2	1/2	2/3	2/3
Sharpness	2/2	2/1	1/1	2/3	3/2	2/2	1/1	1/1	2/3	1/2
Bone details	3/3	2/2	2/2	3/3	3/3	1/1	1/1	1/1	2/3	2/3
Contour visibility (lymphnode)	2/2	2/1	2/1	3/3	3/3	2/2	1/1	1/1	2/3	2/3
Artifact	4/3	3/3	3/3	4/3	4/3	3/3	2/3	2/3	3/3	3/3

Assessment by radiology specialist: In the phantom evaluation, PMPCCT was rated with lower noise levels compared to PMCT, particularly in Br60 across all slice thicknesses. In Br40 reconstruction kernel, the noise reduction of PMCT was rated superior, especially at smaller slice thicknesses. Overall image quality was consistently rated higher for PMPCCT, with advantages also observed in sharpness and bone detail, although differences were rather small. Contour visibility was nearly equivalent between both modalities.

In the decedent analysis, the higher rating of PMPCCT over PMCT remained evident, with a slight advantage in noise reduction, particularly at larger slice thicknesses. The overall image quality, sharpness, and bone detail were consistently rated higher for PMPCCT, with the greatest difference observed in Br60 reconstruction kernel. The artifact formation in PMPCCT and PMCT was assessed as nearly identical, with at most a very slight reduction in PMPCCT. It should be noted that no specialized metal artifact reduction programs inherently available in PCCT were used for our evaluation.

Assessment by radiology resident: Evaluation of the phantom data indicated that PMCT was rated with less noise, particularly at smaller slice thicknesses. However, overall image quality remained in favor of PMPCCT, with a slight advantage in sharpness and bone detail in Br60 reconstruction kernel, while Br40 reconstruction kernel was rated comparable between both methods. Contour visibility was rated slightly higher for PMPCCT across both kernels.

For decedent scans, noise levels were rated nearly equivalent between PMPCCT and PMCT, with both demonstrating increased noise at reduced slice thicknesses. Despite this, PMPCCT was consistently rated higher in terms of image quality, sharpness, bone detail, and contour visibility. As with the radiology specialist’s findings, artifact formation was rated as comparable between both modalities across all slice thicknesses and reconstruction kernels.

## Discussion

In this study, we compared postmortem photon-counting computed tomography (PMPCCT) with conventional postmortem computed tomography (PMCT), evaluating both objective and subjective image quality across several reconstruction kernels and slice thicknesses. Image quality was considered in terms of objective metrics (SNR, CNR, noise) as well as subjective ratings (sharpness, bone detail, contour visibility, artifacts), reflecting the different diagnostic demands of soft tissue (contrast-based) and bone (resolution-based) imaging. Findings under our scanning conditions suggest that PMPCCT offers advantages at moderate slice thicknesses but underperformed in specific scenarios, particularly at ultra-thin slices and in lung imaging. However, its performance is influenced by slice thickness, reconstruction algorithms, and tissue type.

These results are significant as they highlight PMPCCT’s potential to improve postmortem imaging, an area where PMCT often faces challenges. Additionally, the study emphasizes the complexity of postmortem imaging, noting that phantom-based studies do not fully replicate the intricacies of real-world decedent imaging. This underscores the need for further research to optimize PMPCCT for forensic applications. Understanding the nuanced performance of PMPCCT under varying technical parameters can aid in selecting and tailoring imaging protocols for postmortem examinations.

In general, the results show that PMPCCT performed best at slice thicknesses of 0.4 mm, while its advantages diminished at 0.2 mm, possibly due to reduced photon statistics and increased noise [7, 24]. This effect is consistent with some of the previous findings on diminishing returns at extreme resolutions [23, 29].

Aditionally, reconstruction kernels appeared to play a role in image quality differences. The softer Br40 kernel likely helped PMPCCT by reducing noise, whereas the sharper Br60 kernel may have amplified noise, favoring PMCT [6, 28]. This could have been accentuated due to differences in reconstruction algorithms, as PMCT’s ADMIRE 3 is optimized for strong noise suppression [3, 5]. However, the inferior performance of PMPCCT at Br60 in decedent imaging was rather unexpected, as literature generally supports PCCT’s superiority even with sharper kernels [6, 16, 29].

In lung imaging, PMCT outperformed PMPCCT in our study, particularly at thinner slice settings. This result agrees with earlier research showing that PCCT can be prone to elevated high-frequency noise in aerated tissues [20, 27]. Conversely, PMPCCT demonstrated advantages in soft tissue and liver imaging, particularly under Br40 conditions. This is in line with existing literature describing PCCT’s superior soft tissue contrast and resolution [10, 25, 31, 33].

Reconstruction parameters represent an important technical factor in diagnostic assessment. Matrix size directly affects spatial resolution: a 1024 × 1024 matrix, as available on PMPCCT, increases pixel density and improves detail visibility, particularly for bone structures, but also enhances the perception of noise. Slice thickness has a similar bidirectional effect: thinner slices reduce partial volume averaging and improve sharpness but simultaneously increase noise, thereby lowering SNR and CNR. In this study, PMPCCT reconstructions included thinner slices and a larger matrix compared to PMCT, reflecting the standard defaults of each system. These settings may have contributed to the observed improvements in sharpness and bone detail and must therefore be considered potential confounders when interpreting the results.

Additional factors may have influenced performance. Both PMCT and PMPCCT were performed with relatively high tube voltages, as radiation dose is not a limiting factor in postmortem imaging. In clinical settings, PCCT is considered particularly advantageous because it delivers optimal image quality even at lower radiation doses [26, 34]. However, since this benefit is irrelevant in postmortem imaging, the generally higher tube voltages used may have mitigated some of PMPCCT’s expected advantages—an aspect that may warrant further investigation [22, 35]. In this context, it is also relevant that – although scan protocols and scanning conditions were kept as similar as possible between PMPCCT and PMCT – slightly higher CTDI values were ultimately reached in the PMPCCT scans of the decedent. As a result, the associated improved outcomes should be interpreted with caution.

A notable discrepancy emerged between phantom and decedent imaging. Since phantoms consist of homogeneous materials, they minimize scattering effects, whereas decedent scans involve heterogeneous structures that likely led to more complex signal distributions [30, 36]. Prior studies have reported similar deviations between phantom-based and real world imaging outcomes [12, 36], reinforcing the need for caution when extrapolating results. Furthermore, most PCCT studies focus on clinical rather than postmortem imaging so far. Unique postmortem factors, such as the absence of motion artifacts and altered tissue properties, dose independency and body temperature, may influence the comparative performance of PMPCCT and PMCT [30, 37].

The subjective image quality assessment of PMPCCT should be interpreted as indicative rather than conclusive. Due to the small sample size statistical analysis was not feasible. Overall, PMPCCT demonstrated subjective advantages in image quality, sharpness, and bone details while performing similarly to PMCT in contour visibility, noise, and artifact reduction. These findings align with existing literature on PCCT in clinical settings, which consistently highlights improved spatial resolution and contrast [7, 10, 15–18, 30, 35, 36].

The comparable noise perception between PMPCCT and PMCT is consistent with prior research, as overall noise levels depend on reconstruction algorithms and dose settings [28, 29].

The similar performance in artifact formation, especially in the presence of an ICD catheter, is notable. PCCT is suggested to reduce metal artifacts due to its energy-resolving capabilities [11, 15, 17, 36]; however, its effectiveness varies depending on the metal composition and reconstruction techniques used [6, 13, 14, 31]. The similar performance in artifact formation in the presence of an ICD catheter in our study is not very surprising, since no specialized artifact-reducing programs were applied. Follow-up studies would be necessary to objectively assess any potential advantage in this regard.

### Study limitations and future directions

A major limitation of this study is its small sample size, including only one decedent and one phantom, and its controlled experimental conditions. This prevents generalization of the results and precludes any meaningful statistical testing. The study should therefore be regarded as an exploratory pilot investigation, providing first insights into the feasibility and potential of PMPCCT in postmortem imaging. While the findings provide valuable insights, larger studies involving diverse cases and real-world postmortem scenarios are essential to validate the results and account for the variations in tissue composition and imaging challenges in actual forensic investigations.

The discrepancies between phantom and decedent imaging highlight the need for studies conducted under real-world postmortem conditions. Factors such as body temperature, degree of decomposition, and cause of death are likely to influence imaging outcomes and cannot be replicated in phantoms. Although the BMI of the included decedent was within the normal range, differences in body habitus may also affect image quality through variable tissue attenuation. To improve generalizability, future investigations should therefore include cases across a broader BMI spectrum and encompass a wider range of postmortem changes. Such studies will be essential to assess PMPCCT performance under diverse conditions and to optimize imaging protocols for forensic application.

Even though the scanning conditions were kept as consistent as possible, differences in reconstruction parameters between PMCT and PMPCCT, including slice thickness (0.5 mm for PMCT vs. 0.4 mm and 0.2 mm for PMPCCT), reconstruction algorithms, and slightly varying dose metrics, may have influenced image quality, SNR, CNR, and subjective ratings independent of detector technology [2, 6, 7, 16, 20, 23, 28, 29]. These settings were chosen according to standard usage of each system to reflect routine practice and to achieve optimal image quality. Nevertheless, such differences must be considered a potential source of bias. Future studies should therefore investigate harmonized and optimized PCCT reconstruction protocols tailored for postmortem imaging, especially at ultra-thin slices and in complex anatomical regions [6, 16, 28].

ROI measurements were obtained across five consecutive slices per anatomical region by one reader and verified by a second. While this approach improves robustness, reproducibility was not formally assessed and inter- and intraobserver variability remain potential limitations.

Finally, our study was conducted using a 140 kV tube voltage, which may have diminished some of the expected advantages of PMPCCT. Since radiation dose is not a limiting factor in postmortem imaging, future research should explore the effects of lower kV settings to fully exploit the benefits of photon-counting technology, as suggested by clinical applications [22, 26].

Furthermore, it will be essential to investigate the spectral imaging capabilities unique to PMPCCT to differentiate between various tissue types and foreign bodies, which could be especially useful in forensic imaging [6, 10, 11, 13–15, 17, 18, 32, 38].

Optimizing scan protocols and reconstruction algorithms is a critical step toward enhancing PMPCCT’s performance. Such advancements will not only refine the imaging process but also strengthen the diagnostic potential of PCCT in forensic applications.

## Conclusion

PMPCCT may demonstrate advantages in both objective and subjective image quality, with notable improvements in soft tissue imaging, sharpness, and bone detail at moderate slice thicknesses. However, PMCT showed superior performance in lung imaging and at ultra-thin slices. These results may suggest that PMPCCT has promising potential in forensic imaging, while further optimization is needed to fully leverage its strengths across all tissue types and technical settings.

## Key points

1. PMPCCT showed potential advantages over PMCT.
2. Objective measurements indicated variable performance of PMPCCT, with advantages mainly in soft tissues and at medium slice thickness.
3. Differences between phantom and decedent scans underline the limited transferability of model-based studies.
4. Subjective ratings suggested improved sharpness and bone detail with PMPCCT in selected settings.

## Appendix

**Table 4:** Detailed overview of the percent change (PC) in noise, signal-to-noise ratio (SNR), and contrast-to-noise ratio (CNR) between PMPCCT and PMCT across various slice thicknesses, reconstruction kernels, and tissue types. Negative SNR and CNR values indicate poorer objective image quality, while positive values reflect improved objective image quality in PMPCCT

Scan Object	Tissue type	Metrics	PC PMPCCT 1 mm vs. PMCT 1 mm		PC PMPCCT 0.4 mm vs. PMCT 0.5 mm		PC PMPCCT 0.2 mm vs. PMCT 0.5 mm	
			Br40	Br60	Br40	Br60	Br40	Br60
Decendent	Vertebral Body	SNR	34.4	9.2	52.3	-14.1	27.1	-18.8
Decendent	Lung	SNR	-25.7	-54.6	-36.3	-61.7	-50.7	-64.9
Decendent	Muscle	SNR	25.6	-21.3	12.9	-36.1	-11.2	-37.4
Decendent	Blood	SNR	59.3	-2.6	33.4	-23.9	14.7	-22.3
Decendent	Liver	SNR	78.2	24.1	72.2	-8.8	48.9	-7.6
Decendent	Vertebral Body	CNR	16.9	-6.8	29.7	-27.6	11.9	-29.2
Decendent	Lung	CNR	93.7	21.4	71.0	-5.0	44.0	-0.6
Decendent	Muscle	CNR	21.8	-24.7	7.2	-39.1	-12.6	-39.4
Decendent	Blood	CNR	22.6	-24.3	7.6	-38.9	-11.9	-39.0
Decendent	Liver	CNR	41.0	-1.1	40.7	-24.7	17.5	-27.0
Decendent	Overall	Noise	-19.8	21.5	-14.3	53.9	3.4	55.3
Phantom	Vertebral Body	SNR	-9.6	17.4	60.9	54.7	-18.5	-7.5
Phantom	Lung	SNR	-18.1	0.6	47.6	32.9	-25.9	-20.2
Phantom	Chest wall	SNR	-93.4	-94.1	-82.4	-86.8	-82.4	-83.0
Phantom	Mediastinum	SNR	-18.4	3.0	47.0	38.1	-26.1	-17.3
Phantom	Diaphragm block	SNR	-106.5	-100.0	-100.0	-100.0	-100.0	-100.0
Phantom	Sphere 1	SNR	-16.8	37.7	34.1	89.1	-30.0	13.5
Phantom	Sphere 2	SNR	-33.2	-14.4	24.6	22.6	-37.5	-27.4
Phantom	Sphere 3	SNR	-33.7	-15.3	23.2	20.6	-37.8	-28.0
Phantom	Vertebral Body	CNR	-14.6	10.7	53.1	47.0	-23.1	-12.8
Phantom	Lung	CNR	-8.7	64.9	62.7	129.4	-19.6	25.4
Phantom	Chest wall	CNR	-17.3	7.1	48.7	42.7	-25.5	-15.6
Phantom	Mediastinum	CNR	-16.6	8.0	49.9	43.9	-24.9	-15.0
Phantom	Diaphragm block	CNR	-0.5	14.9	10.4	31.1	-31.4	-7.5
Phantom	Sphere 1	CNR	-22.5	17.9	11.8	42.2	-36.1	-6.1
Phantom	Sphere 2	CNR	-34.1	-15.6	24.6	21.4	-37.8	-28.5
Phantom	Sphere 3	CNR	-32.9	-13.2	27.2	25.6	-37.2	-26.7
Phantom	Overall	Noise	24.7	-4	-23.8	-26.6	44.3	18.8
